# Care for patients with cancer and substance use disorders: a qualitative study of oncology team experiences

**DOI:** 10.1007/s00520-025-09181-7

**Published:** 2025-02-04

**Authors:** Sachin S. Kale, Laura J. Rush, Jennifer L. Eramo, Mireille Bitangacha, Sadie Chen, Devon K. Check, Katie Fitzgerald Jones, Jessica Merlin, Ann Scheck McAlearney

**Affiliations:** 1 Division of Palliative Medicine, Department of Internal medicine, College of Medicine, The Ohio State University Wexner Medical Center, 1581 Dodd Drive, Columbus, OH 43210 USA; 2 The Center for the Advancement of Team Science, Analytics, and Systems Thinking in Health Services and Implementation Science Research (CATALYST), College of Medicine, The Ohio State University, 700 Ackerman Road, Suite 4100, Columbus, OH 43202 USA; 3 Department of Population Health Sciences, Duke University School of Medicine, Durham, NC USA; 4 New England Geriatrics Research, Education, and Clinical Center (GRECC), VA Boston Healthcare System, Jamaica Plain, MA USA; 5 Division of General Internal Medicine, CHAllenges in Managing and Preventing Pain (CHAMPP) Clinical Research Center, University of Pittsburgh School of Medicine, Pittsburgh, PA USA; 6 Department of Family and Community Medicine, College of Medicine, The Ohio State University, 700 Ackerman Road, Suite 4100, Columbus, OH 43202 USA

**Keywords:** Cancer, Pain management, Substance use disorder, Opioid use disorder

## Abstract

**Purpose:**

Clinical decisions surrounding cancer care can be complicated by co-occurring substance use disorders (SUDs), particularly with respect to pain management and cancer treatment safety. Yet, few studies have examined oncology teams’ experiences treating patients with co-occurring cancer and SUDs. We therefore sought to understand the perspectives of oncology team providers regarding the challenges they face when caring for patients with cancer and SUDs.

**Methods:**

We conducted a qualitative study to understand the experiences of clinicians who have provided cancer care to patients with concurrent SUD. Questions about substances used focused mainly on non-prescribed opioids and stimulants. Individual interviews were conducted in March–July 2023 by telephone or videoconference, recorded, transcribed verbatim, and analyzed rigorously using thematic analysis.

**Results:**

Twenty-seven individuals were interviewed (15 physicians, 8 advanced practice providers, 2 registered nurses, and 2 social workers). Specialties included medical oncology, hematology, radiation oncology, and gynecology oncology. We identified four themes representing the challenges oncology teams face when caring for patients with cancer and SUD: (1) patients’ unmet social needs; (2) uncertainty about pain management options; (3) implicit biases about patients with SUDs; and( 4) patients’ active substance use.

**Conclusion:**

Oncology teams face many challenges when caring for patients with cancer and SUDs. Understanding these challenges is critical and can both inform the design of interventions for patients with cancer and SUD and guide future research.

## Introduction

The management of patients with concurrent cancer and substance use disorder (SUD) is a challenging clinical issue [1]. This is particularly true of SUDs that increase the risks of opioid-related harms, such as non-prescribed opioids and stimulants, due to the integral role opioids have in the management of cancer-related pain [2, 3]. The prevalence of SUD in patients with cancer ranges between 2 and 35%, depending on the cancer subtype and how SUD is defined [4].

People with SUDs are highly stigmatized, and this stigma can impact their medical care [5]. For example, patients with SUDs are often less likely to receive timely cancer screenings; thus, they are at a higher risk of being diagnosed at an advanced cancer stage compared to their peers [6]. When experiencing cancer-related pain, patients with SUDs are less likely to be offered opioid therapy, leading to poorly controlled symptoms and suffering [7, 8]. People with both cancer and SUDs often have worse outcomes, higher healthcare utilization, and higher mortality rates compared to those patients without SUDs [9–13].

Despite the prevalence of SUDs in cancer, clinicians report feeling unprepared and uncomfortable managing SUDs in the setting of cancer [14]. To date, few studies have investigated the complexity experienced by clinicians caring for patients with SUD and cancer, particularly SUDs such as opioids and simulants that may complicate opioid management for cancer-related symptoms. A deeper understanding of the perceived challenges of caring for this patient population can inform both supportive interventions and avenues for future research. We therefore interviewed oncology team members at our institution to explore clinician perspectives regarding treating patients with cancer and SUD.

## Methods

### Setting

This qualitative study was conducted at a large academic medical center (AMC) in the Midwest. The institution is designated as a National Cancer Institute Comprehensive Cancer Center (NCI-CCC). In 2020, our institution established a Palliative Harm Reduction and Resiliency (PHRR) clinic to provide palliative care to patients with SUD who were receiving cancer treatment [15]. This clinic provides harm reduction and treatment services along with cancer-related symptom management (e.g., pain).

### Sample selection and recruitment

Physicians and oncology team members in medical oncology, gynecologic oncology, hematology, and radiation oncology clinics at the AMC who made at least one referral to the PHRR clinic between January 1, 2021 and March 1, 2022 were eligible to participate in this study. Emails were sent to potential participants that explained the purpose of the study, and interested participants could respond through a Qualtrics link to indicate preferred days and times for an interview. Up to two follow-up emails were sent to those who did not respond. Potential participants included a mix of provider roles (e.g., physicians, advanced practice providers, nurses, social workers) and cancer specialties (e.g., head and neck, gastrointestinal, hematological). Recruitment ended when saturation was reached in the data collection [16]. A $25 Amazon gift card was offered for interview participation.

### Development of interview guide

A semi-structured interview guide was developed with input from the study team. Topics included providers’ experiences with patients with SUD including opioid and stimulant use disorders who had cancer and needed pain management. Additional questions focused on providers’ backgrounds and training in palliative care and substance use disorders/addiction and their experience prescribing opioids to manage cancer-related pain. The interview guide was pilot tested before refinement and use in the study.

### Data collection

Individual interviews were conducted between March and July 2023. Research team members conducted interviews remotely via videoconference or telephone depending on interviewee preference. Interviewers did not know nor have any prior relationship with the participants. Participants provided verbal consent prior to the interview. Interviews were recorded, transcribed verbatim, and de-identified.

### Data analysis

Transcripts were analyzed using both deductive and inductive thematic analysis [17]. An initial codebook was developed based on the topics covered by questions in the interview guide. Four members of the research team coded a single transcript using the initial coding dictionary and then met to compare coding decisions. Discussion among the group informed refinement of the coding dictionary. This team divided the remaining transcripts for independent coding, meeting weekly to discuss concerns, discrepancies, and emergent themes as the coding proceeded. Our work follows the Standards for Reporting Qualitative Research [18]. We used ATLAS.ti 23.1.1 qualitative software (ATLAS.ti Scientific Software Development GmbH, Berlin, Germany) to support the coding and analysis process.

## Results

We completed interviews with 27 providers: 15 physicians, 7 nurse practitioners, 2 registered nurses, 2 social workers, and 1 physician assistant. Interviews ranged in length from 15:07 to 58:06 min with an average time of 33:27 min. Nearly two-thirds of our participants were female, and the most common specialty was medical oncology. Most interviewees had been practicing for 15 years or less. None of the participants had certifications in addiction medicine. See Table 1 for participant characteristics.

**Table 1:** Interviewee characteristics

Characteristic	*n* (%)
Provider role (*N* = 27)	
Physician	15 (56)
Advanced practice provider	8 (30)
Staff registered nurse	2 (7)
Social worker	2 (7)
Sex	
Male	10 (37)
Female	17 (63)
Cancer specialty	
Gynecologic oncology	4 (15)
Hematology	3 (11)
Medical oncology	18 (67)
Radiation oncology	2 (7)
Years of experience	
< 5	5 (19)
6–15	14 (52)
16–25	4 (15)
> 25	4 (15)

Participants described various issues they face providing optimal cancer treatment for patients with cancer and SUD. We characterized four themes around challenges providing this care: (1) patients’ unmet social needs; (2) uncertainty about pain management options; (3) implicit biases about patients with SUD; and (4) patients’ active substance use. Each theme is presented in greater detail below with additional quotes supporting the characterization of these themes presented in Table 2.

**Table 2:** Providers' perspectives about challenges caring for patients with cancer and concurrent substance use disorder, by theme

Theme	Verbatim provider quotations
Patients’ unmet social needs	“They’re overwhelmed with the new diagnosis, and if these are, like, chemoradiation patients for instance…it’s a very difficult treatment. And it’s almost like a full-time job… And a lot of our patients don’t always have the financial or social support to help get them through this treatment…. A lot of patients are really overwhelmed.” (03)
	“When you look at Maslow’s Hierarchy of Needs, these folks are all on the bottom of that pyramid, and they are looking for those things. Just everyday things. They will not come into clinics for chemo[therapy] because…they’re looking for a social worker to help them with housing, to help them with food, to help them with…all the basic things that that you need.” (09)
Uncertainty about pain management options	“I don’t think that the pain management sometimes can be achieved if they are currently using (illegal substances), because of fear of them causing more harm than good, and even death.” (11)
	“From my experience…it’s harder to get their pain under control. …it’s something that you don’t want to discriminate or be judgmental and say…they just want higher pain medicine because they…have that addiction problem. But I do think that it’s more challenging to treat their pain. And some of these patients are on Suboxone or other things like that…can make it more challenging to get their pain controlled.” (20)
Implicit biases about patients with SUD	“I’m a white man of a certain age, and I have my certain biases that I try to overcome…. I’m, like, ‘Oh, gosh, here’s a drug abuser…who I know, for example, by history. I know this guy has a history of drug use, and, oh, this going to be a disaster.” (28)
	“It’s a very difficult experience for them because…we have a lot of biases…. I’m definitely a little bit more careful if I know they have substance abuse. So, I am going to limit what I give. I’m going to try to use non-narcotic medications and our other avenues of pain management for them first to see whether they get better…So, even my prescribing, my approach to their pain management does change, but I try my best that I’m still addressing their issues.” (19)
Patients’ active substance use	“If they’re noncompliant with follow-ups, then that would really push me toward…limiting their systemic therapies for this cancer. Because…there’s no way for you to monitor the side effects. And…to me, that’s…pretty dangerous.” (25)
	“I would never jeopardize not giving someone standard of care treatment, but I think that there’s certain, like, nuances that I sometimes modify… For example, I have a lady in my practice that uses cocaine, and she has recurrent [type] cancer, and she is on immunotherapy…given every three weeks. But we can also give it every six weeks and the outcomes are equivalent… So I actually then switched her to six-week dosing schedule to try and make it easier for her to come. And I actually think that’s helped… I would never give someone substandard treatment because of their substance abuse disorder. (07)”

### Patients’ unmet social needs

Participants noted that many patients with SUD had significant socioeconomic needs in addition to their substance use and this posed additional challenges during cancer treatment. For instance, patients with SUD were noted to have had co-occurring housing instability, lack of social support, transportation needs, and poor financial resources prior to their cancer diagnosis; and these needs were exacerbated when they received a cancer diagnosis and initiated cancer treatment. As one provider commented,

Their support systems are limited, if existing. The stability isn’t there that other folks may have. Their abilities to engage are limited…and, usually, unfortunately, there's unmanaged trauma, mental health that coincides. So, it’s multifactorial when you look at what you’re treating. Not just treating the substance abuse, it's treating the mental health, it’s treating the social determinants of health, it’s treating the instability. So, it takes a lot of work to care for someone in that place. (08)

Similarly, challenges associated with patients’ social situations were frequently mentioned, as another provider reflected,

“I can think of several people in our population that they are financially, they just don't have it all together…now they’re ill. They can't work. They may or may not have kids or their support system are not really there…are just not available to help them out whether physically, like in the vicinity, or they themselves have responsibilities that take them away…or they’re pretty isolated. They just kind of come into town by themselves and they have only one other partner and that partner is also in the same boat, maybe not with cancer, but financially, and they, too, have like anxiety and depression disorder or some kind of drug disorder.” (10)

### Uncertainty about pain management options

Many study participants described the tension they experienced when trying to relieve pain while also prioritizing patient safety. In addition, providers commented that they worried about the ethical considerations of not prescribing opioids to a patient with SUD when it would otherwise be indicated as well as the risks of patients using non-prescribed substances to treat uncontrolled pain. One participant reflected,

There’s a big knowledge deficit in terms…of…standard care…So, then it becomes how can we treat this pain without…opiates. And…now on the flip side…even though there’s a hesitancy…for me to prescribe…if I have somebody that’s got this fungating mass coming off of their [location on the body]…I know that if I don’t provide them with pain relief, that they are going to go out and get heroin…or fentanyl off the street…So, there have been times that I’ve been…nervous about this and it makes me uncomfortable that I know that they’ve got this history…[but] I still feel ethically…that I need to…prescribe opiates.” (04)

Another respondent similarly described this wariness, emphasizing the possible consequences for both the patient and prescriber:

It’s a real double-edged sword.… It’s extremely sad for the patient, and at the same time, it’s also difficult for us because we could easily go wrong, and we could cause extreme harm to the patient. So…we…rather sometimes not…provide as much pain relief because we are worried…that there could be a really negative impact on the patient… (19)

At the same time, interviewees shared that even when they prescribed opioids, they might delay prescribing or prescribe in lower doses than they may have otherwise in order to limit the potential for patients to overdose on the prescribed medications. As one noted,

The concern over safety with prescribing, like, opioids and someone who might be using illicit substances or…abusing opioids…So…you may tend to…either not give them meds as quickly or maybe [at] lower doses than what you typically would just out of concern for their safety. (18)

While most participants’ comments focused on patients with active SUDs, some also noted concerns about safe pain management in patients with a prior history of a SUD, especially for those patients who had a previous history of opioid use disorder. As one noted,

Opiates, they’re not a great…long-term pain regimen. And they’re going to continue to…get some kind of dependence on it physiologically. And so, I worry that if I’m starting to give them opiates again, that I’m going to…regress [them] back to their addiction (04).

### Implicit biases about patients with SUD

Some clinicians’ comments acknowledged the need for awareness about implicit biases when interacting with and caring for patients with cancer and SUD. For instance, one provider described subconsciously questioning a patient’s motives for requesting pain medication:

I think there’s also, like, the challenge of implicit bias from us as providers in caring for patients that have substance abuse disorders. Like…are they really having pain? Do they really need these much meds? Oh…are they going to use half of these and then take the rest and sell them? (07)

Participants also discussed challenges that could arise when patients have pain for longer than anticipated, particularly after cancer treatment, and discerning whether this is related to a desire for continued opioids. A different provider reflected,

For instance, patients who get chemoradiation…usually the pain should go away…And patients with substance abuse…they [may] still complain of some pain. So, it’s very difficult…to find out if it’s real pain. (11)

Another clinician noted that patients might withhold information about their drug use history to avoid being stigmatized and undertreated, explaining,

And sometimes there’s misconceptions, misbeliefs, misunderstanding about folks who are in the space of substance misuse, so there can be bias or implicit bias that weighs in on that from providers, too. And that can also create challenges ‘cause patients are not wanting to share that information that they have had a past history because they’re concerned that it will compromise their care or their ability to get care that other patients might be offered in the space of pain management. (08)

### Patients’ active substance use

Finally, providers noted that when patients were actively using substances, this could impact both patient care and outcomes. One clinician explained how patients’ substance use could impair their ability to keep appointments, and, consequently, such patients might not receive the optimal doses of treatments such as chemotherapy or radiation:

If there’s somebody with a substance use disorder, somebody who’s not showing up…we are really worried that if we give them something that causes their counts to drop, that they will not make it back into the hospital for labs, blood, or platelets…They may put them on a regimen that it’s not necessarily the best regimen for their cancer, but at least it will not kill them… (09)

Another clinician explained that treatment plans occasionally needed to be modified for safety reasons:

If they use multiple substances, then you have to worry about the potential risk for drug-to-drug interactions… That is still dangerous especially…if they’re taking opiates concurrently with that. If they’re using cocaine,…that presents a different set of problems. (25)

## Discussion

Our study is among the first to provide qualitative data describing the experiences of oncology teams in caring for patients with cancer and SUDs such as nonmedical opioid and stimulant use. We characterized several themes that providers reported impacted their ability to provide optimal care for patients with cancer and concurrent SUDs. Notably, our respondents reported that many patients with SUDs face challenges related to financial and social hardships that can limit their ability to adhere to cancer treatment. Patients with cancer are more likely to face debt and file for bankruptcy compared to those without cancer due, in part, to the loss of job opportunities as well as from costs associated with cancer treatment [19]. Many patients with SUDs are already disproportionately affected by depleted financial resources and unmet social needs, irrespective of a cancer diagnosis [20, 21]. One study reported that veterans with cancer and SUDs were more likely to experience homelessness than those without SUDs [22]. In our study, clinicians perceived that patients with cancer and SUDs often suffer from financial constraints as well as transportation limitations that may drastically impair their ability to adhere to cancer treatment. This suggests that patients with SUDs should be proactively screened for unmet social needs that may impact cancer outcomes, including income, insurance, and availability of transportation.

The challenges of providing safe and effective pain management for patients with SUDs, specifically opioid use disorder, have been increasingly recognized [1, 23–26]. Many of our study respondents reported they were uncertain about how to effectively prescribe opioids for the management of cancer pain for without risking drug relapse or overdose. This uncertainty is important because opioids remain the gold standard of care for moderate to severe cancer pain [27]. Limiting opioids, even if due to concerns about drug overdose or misuse, risks the mismanagement of pain. Research has established that poorly treated cancer-related pain is associated with several negative outcomes, including higher healthcare utilization, decreased functional status, and increased emotional distress [28–32]. Strategies and models of care have emerged in recent years that may provide safer pain management for patients with concurrent SUDs. For instance, buprenorphine can be a highly effective option to treat pain, and multidisciplinary collaboration, including with palliative medicine, can provide additional expertise in pain management to care teams [15, 24, 26, 33, 34]. Utilizing these strategies may help address some of the challenges reported by our study participants.

Clinicians in our study also commented about the impact that implicit bias and stigma can have on caring for patients with cancer and concurrent SUD. Indeed, several studies have found that bias and stigma from healthcare professionals can have detrimental impacts on patients with SUDs [35, 36]. Our study participants noted the potential for bias particularly in relation to decisions about prescribing opioids to treat severe pain. Although not directly addressed, it is possible that such biases could also impact decisions about a patient’s ability to adhere to and tolerate cancer treatment. Acknowledging this challenge suggests opportunities for oncology clinicians to engage in programs designed to reduce bias and stigma in healthcare [37, 38]. Additionally, new requirements from the Drug Enforcement Agency regarding training in opioid use disorder may help to improve knowledge of SUDs and potentially expand access to medications for opioid use disorder [39].

Clinical decisions about appropriate cancer treatment options are among the most challenging and impactful decisions that providers face. Clinicians in our study acknowledged that the presence of an active SUD may at times lead them to modify their cancer treatment compared to what is typically administered to maintain patient safety. Providers were cognizant of the importance of critically evaluating each clinical situation. Unfortunately, the ability of patients to access SUD treatments while undergoing cancer treatment can be impeded by patient debility and regulations around some SUD treatment programs [40]. Potential risks from SUDs included acute toxicities from nonmedical substance use such as overdose or infection, the potential impact of an SUD on a patient’s ability to tolerate treatment side effects, and the risk of treatment nonadherence. Limited empirical evidence exists to guide clinician decisions about the risks of concurrent SUDs on cancer treatment. Topics for future exploration include risks of concurrent cancer treatment based on type and severity of SUD, as well as whether the addition of resources that are tailored to reduce the harms of substance use or treat SUD may improve perceived safety in prescribing cancer treatments.

This study has limitations. First, it was conducted at a single institution in a Midwestern state that was particularly hard-hit by the opioid crisis. Second, clinicians in our study had previously cared for at least one patient with SUD, though none had addiction certification. It is possible that interviewing oncology clinicians who had never cared for a patient with SUD would provide additional insight about these challenges. Third, our interview questions focused on opioid and stimulant use disorders, and thus additional study will be needed to consider other SUDs such as alcohol.

## Conclusion

Our qualitative study shows oncology teams deeply engaged in caring for patients with cancer and concurrent SUDs while navigating many challenges in providing optimal cancer care to this population. These challenges include addressing the unmet social needs of patients, balancing analgesia with safety concerns, recognizing and responding to implicit bias, and providing safe cancer treatment in the setting of active SUD. Early involvement of social work, providing access to palliative medicine and addiction management services, implementing training on implicit bias, and disseminating evidence-based guidance on the risks of cancer treatment for patients with SUDs are examples of approaches that can provide additional support for oncology teams caring for this complex patient population. Future research is needed on the development and implementation of such interventions to help address these important challenges and improve cancer care for this vulnerable population.

## Data Availability

The data that support the findings of this study are not available due to reaons of privacy and confidentiality.
